# Maternal Pregnancy Intention and Professional Antenatal Care Utilization in Bangladesh: A Nationwide Population-Based Survey

**DOI:** 10.1371/journal.pone.0157760

**Published:** 2016-06-16

**Authors:** Md. Mosfequr Rahman, Md. Mizanur Rahman, Md. Ismail Tareque, Jannatul Ferdos, Syeda S. Jesmin

**Affiliations:** 1 Department of Population Science and Human Resource Development, University of Rajshahi, Rajshahi, Bangladesh; 2 Department of Global Health Policy, School of International Health, The University of Tokyo, Tokyo, Japan; 3 Department of Sociology, University of North Texas at Dallas, Dallas, Texas, United States of America; Royal Children's Hospital, AUSTRALIA

## Abstract

**Objective:**

To investigate the association between maternal pregnancy intention and professional antenatal and delivery care utilization.

**Methods:**

Our data were derived from the 2011 nationally representative Bangladesh Demographic Health Survey. We included antenatal and delivery care utilization data of the most recent live births for women for the previous three years (n = 4672). We used multilevel logistic regression models to assess the relationship between pregnancy intention and use of professional antenatal and delivery care, with adjustment for potential confounding variables.

**Results:**

Approximately 13% and 16% of children were reported by their mothers as unwanted and mistimed at the time of conception, respectively. Among the women, 55% received at least one professional antenatal care service; 21% received four or more professional antenatal services, while 32% were attended by professionals during deliveries. Mothers of children whose pregnancies had been unwanted had a greater risk for not seeking professional antenatal and professional delivery care than those whose pregnancies had been wanted [1≥ ANC from professionals: AOR: 0.66; 95% CI:0.51–0.93; 4≥ ANC from professionals: AOR:0.56; 95% CI:0.37–0.84; and delivery care from professionals: AOR: 0.70; 95% CI:0.50–0.97]. Women who were married after age 18, had secondary or higher level of education, and were from the wealthiest households were more likely to utilize antenatal and delivery care.

**Conclusion:**

Unwanted pregnancy is significantly associated with lower utilization of professional antenatal and delivery care services in Bangladesh. Reducing unwanted births and promoting access to professional antenatal and delivery care are crucial for achieving the Sustainable Development Goals (SDGs) 3 in Bangladesh.

## Introduction

Worldwide, approximately 830 women die from preventable causes related to pregnancy and childbirth every day; 99% of these deaths take place in developing countries [1]. It is evident that this high rate of maternal deaths in developing countries is associated with inadequate and poor-quality maternal health care [2, 3]. Although the World Health Organization (WHO) recommends that under normal circumstances a pregnant women should have at least four antenatal care visits, in the low-income countries where women experience widespread gender disparities, their access to and use of antenatal care (ANC) are limited. Many factors are known to contribute to women’s lower use of antenatal care in the developing countries, including cultural norms [4], women’s decision making power and status [5], and women’s access to information and services [6]. Intimate Partner Violence (IPV) is another important factor that has potential impact on utilizing maternal health care services. Earlier literature, including a study in Bangladesh, documented that women who had experienced IPV were less likely to utilize ANC [7, 8]. The explanation to the lower ANC use by women in abusive relationships is that pregnancies for these women could have been unintended [9–11], which in turn may lead to lower motivation to seek ANC, or that the pregnancies could have resulted in non-live births (e.g. miscarriage, induced abortion, or termination of pregnancy) [9, 11–14]. The understanding of the factors which affect women’s maternal care practices in the context of their sociocultural environment is essential to develop effective public health programs and interventions for reducing maternal and neonatal deaths.

Pregnancy intention, a woman’s desire for pregnancy prior to or at the time of conception, is an important concept in reproductive health research and practice. The association of unintended pregnancies, pregnancies that are reported to have been either unwanted (i.e., they occurred when no children, or no more children, were desired) or mistimed (i.e., they occurred earlier than desired) [15], and inadequate antenatal care has been documented in the literature [16–18]. Women who experience unwanted pregnancies are at greater risks of delaying antenatal care or not using care [19–24]. Several studies have shown associations between sexual abuse and unwanted pregnancy [9, 10, 25]. This in turn may result in women being reluctant to seek antenatal care. However, Marston and Cleland found that birth order was a more powerful predictor of antenatal care use, than unintended pregnancy [26]. An Indian study found lower odds of mistimed pregnancies being delivered by a trained professional than wanted pregnancies, but did not find an association for unwanted births [27]. This variation could be due to complexity in measuring pregnancy intention among women [28]. Pregnancy intention involves human emotional and psychological factors which may vary in different times and socio-economic strata of a society. Given these inconsistent findings, there is a need for further investigation of factors associated with utilization of antenatal care by the women whose pregnancies are unintended, particularly in countries where unintended pregnancies are common, and abortion is illegal.

In Bangladesh, despite a significant increase in modern contraceptive use over the last two decades, from 36% in 1993–94 to 54% in 2014 [29], the rate of unwanted births remained almost the same. In the years 1993–94 and 2011, 13% of births were reported as unwanted [30]. Although the maternal mortality ratio (MMR) decreased from 322 deaths per 100,000 livebirths in 1998–2001 to 194 deaths per 100,000 livebirths in 2007–2010 with an annual rate of decrease of 5.6% [31], more than one third (36%) of pregnant women still do not receive any professional ANC services, and only one third (31%) of pregnant women received a minimum of four ANC services from professionals or non-professionals health care providers [29]. Only four in ten deliveries (42%) in Bangladesh are attended by professionals. To our knowledge, no comprehensive studies have been undertaken to assess the effects on unintended pregnancies on women’s antenatal care-seeking practices in Bangladesh. To address this gap we examined the association between pregnancy intention and antenatal and delivery care seeking behaviors among women in Bangladesh drawing on a large nationally representative data set.

## Methods

### Data

We used data from the 2011 Bangladesh Demographic Health Survey (BDHS), a cross-sectional nationally representative sample of childbearing aged women. The BDHS sample was collected using a two-stage stratified cluster sample of households. In total 18,222 eligible women aged 12–49 years were selected to participate in the survey; 17,842 of these women completed interviews, yielding a response rate of 98%. Respondents provided information about themselves, their children, and their households. Some information about women was not included in the 2011 BDHS such as domestic violence, which was present in the previous 2007 BDHS [32].The data of the 2011 BDHS used in this study was obtained from the MEASURE DHS archive. Details of data collection and management procedures are described elsewhere [30]. The data set of this study was restricted to last-born children who were aged 35 months or younger at the time of the survey. The rationale for selecting the last-born children within the three years preceding the survey was to minimize recall bias and underreporting of unwanted births. Therefore, the final sample of 4,672 women was used for current analysis.

### Outcome

We considered two indicators as our primary outcome of interest: utilization of professional antenatal care, and utilization of professional delivery care. Based on the definitions used in the 2011 BDHS [30], we defined utilization of professional ANC as utilization of services from a qualified doctor, nurse, midwife, paramedic, family welfare visitor, community skilled birth attendant, medical assistant, or sub-assistant community medical officer. The utilization of professional antenatal care was measured by the frequency of ANC visits from professionals. It was categorized into two groups consisting of (i) women receiving at least one professional ANCvs no ANC (1≥ professional ANC), and (ii) women receiving four or more professional ANC (4≥ professional ANC). The other measure, professional delivery care, referred to birth that was assisted by a qualified doctor, nurse, midwife, paramedic, family welfare visitor, or community skilled birth attendant.

### Predictor variable

Maternal pregnancy intention was the main predictor variable in this study. In the BDHS, maternal pregnancy intention was measured by a series of questions that asked women to recall their feelings at the time of conception for each baby born within the past five years. Women were asked whether the pregnancy had been planned (desired at that time), mistimed (the pregnancy occurred earlier than desired), or unwanted (the pregnancy occurred when no children, or no more children, were desired).

### Confounding variables

A number of individual and household characteristics were included in the analysis as potential confounding factors which have been shown to be associated with receiving ANC in previous studies [19, 33–37]. These include age of mothers (classified as 13–24, 25–34 or 35–49 years), age at first marriage (less than 18 years, and 18 years or higher), maternal education (no education, primary education with less than or equal to 5 years of schooling, and any secondary or higher education with 6 years of schooling and above), age difference of the spouses (<5 years, 5–10 years or ≥11 years), deciding own health care (participated or not participated), current work status (working or not working), order of birth (1^st^ order and 2^nd^ or higher order), place of residence (urban and rural), and region. The wealth variable categorized respondents into quintiles according to the household’s score on the DHS wealth index, which is based on the household’s durable consumer goods, housing quality, and water and sanitation facilities [38]. However, due to unavailability of the IPV data in the 2011 BDHS, we were unable to include IPV in our analysis, which appears to be directly associated with receiving ANC and delivery assistance [7].

### Ethics statement

We obtained permission from the MEASURE DHS to download and analyze the 2011 BDHS data-set for our study purpose. The data were originally collected by the Macro, Calverton, USA. The 2011 BDHS data collection procedures were approved by the ORC Macro-institutional review board. The protocol of the survey was reviewed and approved by the National Ethics Review Committee of the Bangladesh Ministry of Health and Family Welfare.

### Statistical analyses

We used χ^2^ tests to identify bivariate associations between maternal pregnancy intention and other individual and household characteristics, and utilization of professional antenatal and delivery care services. The 2011 BDHS used multistage stratified cluster sampling technique that was based on nested sources of variability such as individuals who were nested in households and households that were nested in communities. In order to account for this, this study used multilevel logistic regression models with a random intercept at the household and the community levels. More valid results in the multilevel analysis are produced when lower levels are nested within higher levels [39, 40]. None of our variables had any missing values. Both the univariate and multilevel regression analyses were performed taking into account the probability sample design. The *svy* commands were applied in univariate and bivariate analyses, and for the multilevel analysis to account for probability weight that was proposed by Rabe-Hesketh and Skrondal [39]. All statistical analyses were conducted using Stata version 13.1/MP (StataCorp, LP, College Station, Texas, USA).

## Results

**Fig 1:**
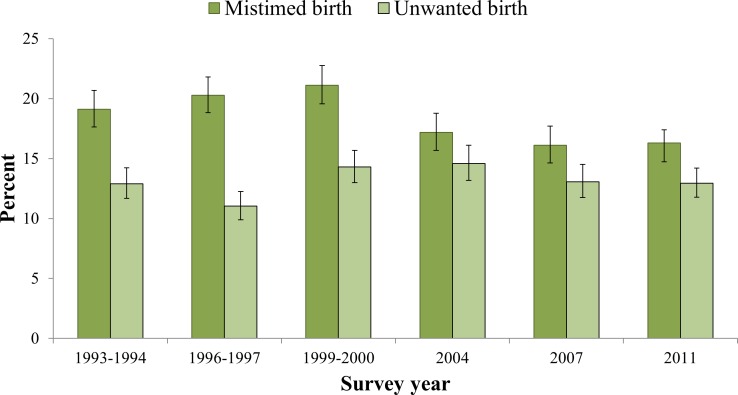
Unintended conception of last child born to women in the preceding three years by BDHS year, 1993–2011.

**Fig 2:**
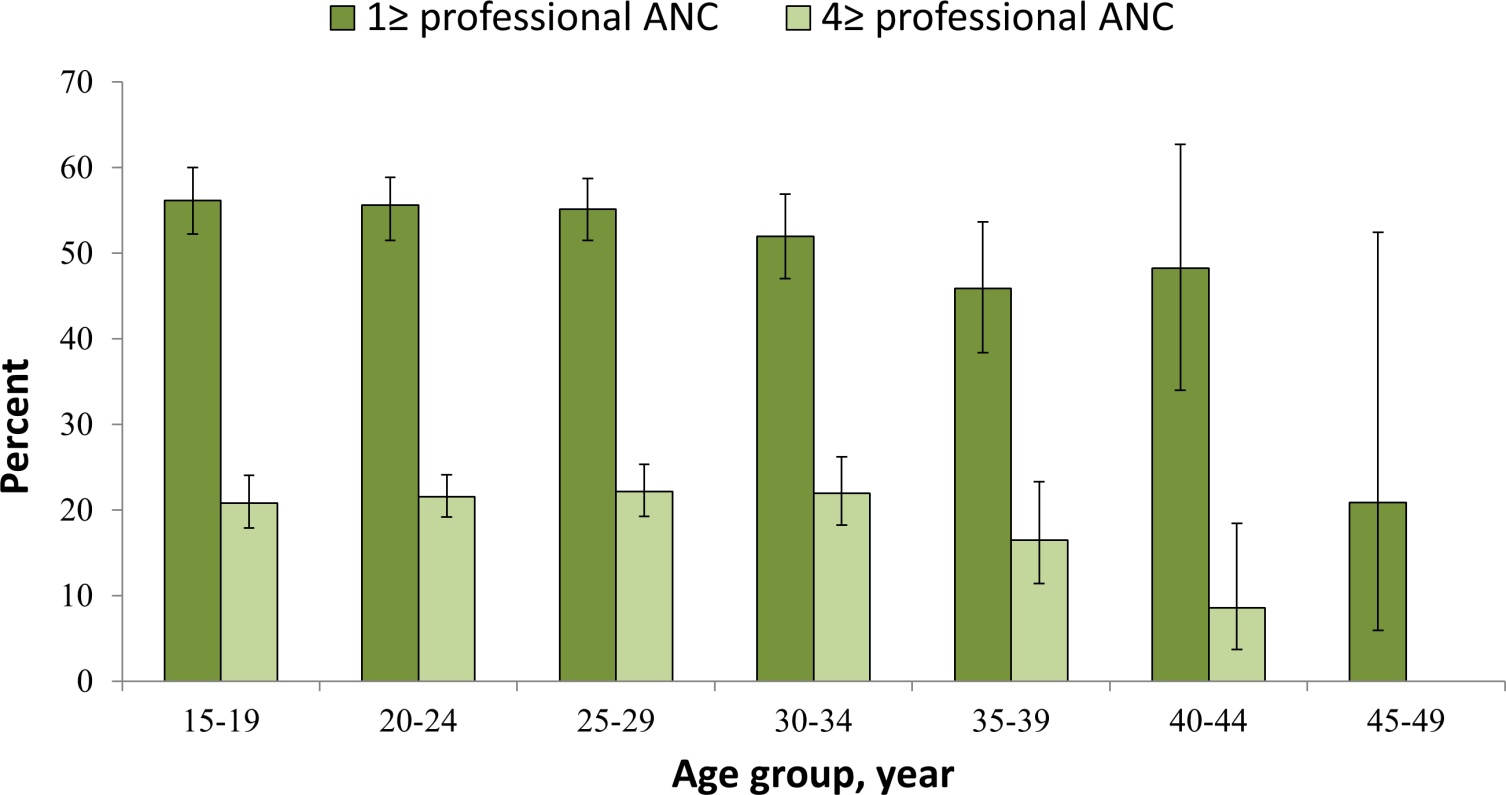
Age-specific antenatal care from medically trained providers, BDHS 2011.

Table 1 shows the sociodemographic and household characteristics of the sample. A majority of the respondents were young aged, 13–24 years (57.1%) and married before reaching their 18^th^ birthday (76.5%). 17.6% of respondents have no education, 42.2% did not participate in making decisions about their health care, 77.0% of respondents were from rural background and more than one-third (36%) of births were reported as first order births. Approximately 13% and 16% of the children were reported by their mothers as unwanted and mistimed at the time of conception, respectively. Unwanted conception of the last born child ranged between 11.03% in 1996–97 to 14.98% in 2004 (Fig 1). About 55% of the women received at least one professional ANC service; 21% received four or more professional ANC services, and 32% deliveries were assisted by professionals (Table 2). Both utilizations of ≥1 ANC services and ≥4 ANC services from professionals decreased with increasing age of the women (Fig 2).

**Table 1:** Characteristics of women aged 13–49 years who had at least one birth in three years preceding the survey, BDHS 2011.

Characteristics	Number (n = 4672)	Percent^a^	95% CI
**Intendedness at conception**			
Intended	3329	71.0	69.3–72.7
Mistimed	766	16.0	14.7–17.4
Unwanted	577	12.9	11.8–14.2
**Individual**			
**Age of mothers, years**			
13–24	2642	57.1	55.3–58.9
25–34	1745	37.3	35.5–39.0
35–49	285	5.6	4.9–6.4
**Age at first marriage, years**			
<18	3475	76.5	74.8–78.1
≥18	1197	23.5	21.9–25.2
**Maternal education**			
No education	774	17.6	15.8–19.5
Primary education	1369	30.1	28.6–32.0
Secondary or higher	2529	52.4	49.7–55.0
**Age difference of the spouses, years**			
<5	905	19.7	18.4–21.2
5–10	2357	50.9	49.2–52.6
≥11	1410	29.4	27.7–31.0
**Deciding own health care**			
Participated	2767	58.4	55.7–59.9
Not participated	1905	42.2	40.8–44.3
**Current work status**			
Currently not working	4246	92.1	91.0–93.0
Currently working	386	8.0	7.0–9.0
**Order of birth**			
First	1721	36.1	34.4–37.9
Second and above	2951	63.9	62.1–65.6
**Household**			
**Place of residence**			
Urban	1481	23.0	21.6–24.4
Rural	3191	77.0	75.7–78.4
**Socioeconomic status**			
Poorest	1001	22.8	20.6–25.2
Poorer	877	19.8	18.3–21.3
Average	893	19.8	18.2–21.4
Richer	948	19.6	18.0–21.3
Richest	953	18.1	16.4–19.9
**Region**			
Barisal	526	5.6	5.1–6.2
Chittagong	942	23.3	21.8–24.8
Dhaka	755	30.5	28.8–32.2
Khulna	552	9.5	8.7–10.3
Rajshahi	593	13.3	11.9–14.8
Rangpur	593	10.5	9.6–11.5
Sylhet	711	7.4	6.8–8.0

BDHS, Bangladesh Demographic Health Survey; CI, Confidence interval

^a^In estimating percentages, the complex survey design and sampling weights were taken into account.

**Table 2:** Descriptive statistics of women by receiving ANC and delivery care from professionals, BDHS 2011 (n = 4672).

Characteristics	≥1 professional ANC, %^a^, (95% CI)	≥4 professional ANC, %^a^ (95% CI)	Professional delivery care, %^a^ (95% CI)
**Intendedness at conception**			
Intended	58.3 (55.7–60.9)	23.4 (21.4–25.5)	34.81 (32.50–37.19)
Mistimed	50.9 (46.6–55.2)	21.3 (18.0–25.1)	30.51 (26.44–34.91)
Unwanted	38.8 (34.1–43.8)	9.0 (6.7–12.1)	17.95 (14.68–21.75)
^**b**^**P-value**	<0.01	<0.01	<0.01
**Individual**			
**Age of mothers, years**			
15–24	55.8 (53.0–58.6)	21.3 (19.3–23.5)	31.8 (29.4–34.3)
25–34	54.1 (50.9–57.3)	22.1 (19.6–24.9)	32.9 (29.8–36.1)
35–49	45.2 (38.7–51.9)	13.7 (9.7–18.9)	27.0 (21.4–33.5)
**P-value**	0.01	0.02	0.25
**Age at first marriage, years**			
<18	49.8 (47.2–52.4)	17.4 (15.7–19.2)	26.9 (24.8–29.1)
≥18	70.2 (66.7–73.5)	33.7 (30.2–37.2)	48.4 (44.3–52.4)
**P-value**	<0.01	<0.01	<0.01
**Maternal education**			
No education	26.2 (22.6–30.0)	5.8 (4.2–8.1)	13.2 (10.6–16.4)
Primary education	40.7 (37.1–44.4)	10.5 (8.6–12.8)	19.9 (17.3–22.9)
Secondary or higher	72.1 (69.9–74.2)	32.5 (30.1–34.9)	45.1 (42.5–47.8)
**P-value**	<0.01	<0.01	<0.01
**Age difference of the spouses, years**			
<5	52.9 (48.7–57.1)	23.2 (19.8–26.9)	33.3 (29.6–37.3)
5–10	53.9 (51.1–56.7)	20.0 (18.0–22.2)	31.4 (28.9–34.0)
≥11	56.8 (53.2–60.3)	21.8 (19.2–24.8)	31.9 (28.6–35.5)
P-value	0.23	0.21	0.66
**Deciding own health care**			
Participated	56.4 (53.8–58.9)	22.5 (20.5–24.7)	33.5 (31.1–35.9)
Not participated	52.5 (48.8–54.6)	19.7 (17.0–22.0)	29.8 (27.0–32.8)
P-value	0.03	0.06	0.02
**Current work status**			
Currently not working	54.5 (52.0–57.0)	21.1 (19.3–23.0)	31.6 (29.5–33.9)
Currently working	55.4 (52.2–56.9)	22.0 (17.7–27.0)	35.2 (30.1–40.7)
P-value	0.76	0.71	0.20
**Order of birth**			
First	66.1 (63.1–69.0)	29.5 (26.8–32.3)	43.7 (40.8–46.7)
Second and above	48.1 (45.4–50.8	16.5 (14.8–18.4)	25.3 (23.0–27.6)
P-value	<0.01	<0.01	<0.01
**Household**			
**Place of residence**			
Urban	74.3 (70.2–77.9)	40.55 (36.7–44.6)	54.1 (49.6–58.5)
Rural	48.7 (45.9–51.5)	15.41 (13.6–17.5)	25.3 (23.1–27.7)
P-value	<0.01	<0.01	<0.01
**Socioeconomic status**			
Poorest	30.3 (27.1–33.8)	6.0 (4.5–8.0)	11.7 (9.6–14.3)
Poorer	39.6 (35.4–43.9)	9.7 (7.7–12.2)	17.9 (15.0–21.3)
Average	54.2 (50.5–57.9)	17.0 (14.4–20.1)	28.4 (25.1–31.8)
Richer	68.1 (64.3–71.7)	27.7 (24.3–31.3)	43.7 (40.0–47.5)
Richest	87.3 (84.5–89.8)	50.4 (46.1–54.6)	63.9 (59.5–68.0)
P-value	<0.01	<0.01	<0.01
**Region**			
Barisal	50.8 (45.4–56.2)	20.6 (16.5–25.5)	28.3 (23.1–34.0)
Chittagong	55.1 (49.2–60.9)	18.4 (14.7–22.8)	30.2 (25.6–35.3)
Dhaka	54.5 (49.7–59.2)	22.1 (18.7–25.9)	32.1 (27.8–36.6)
Khulna	65.4 (60.0–70.4)	30.2 (25.5–35.4)	48.9 (43.5–54.3)
Rajshahi	56.1 (49.4–62.5)	18.0 (14.0–22.6)	31.2 (25.5–37.5)
Rangpur	49.6 (44.3–54.9)	25.4 (20.4–31.2)	28.1 (23.6–33.1)
Sylhet	46.7 (40.2–53.4)	14.6 (10.9–19.3)	24.7 (20.4–29.6)
P-value	0.01	0.01	<0.01
**Total**	54.6	21.2	31.9

ANC, Antenatal care; CI, Confidence interval

^a^In estimating percentages, the complex survey design and sampling weights were taken into account.

^*b*^*P*-values were derived using a *χ*2 test.

Table 2 shows the bivariate association between pregnancy intention and sociodemographic and household variables, and utilization of professional ANC and delivery care services. Mothers of children whose pregnancy had been unwanted had utilized lower levels of professional ANC and delivery care services than mothers of children whose pregnancy had been intended (≥1 ANC from professionals: 38.81% vs 58.30; ≥4 ANC from professionals: 9.03% vs 23.36%; delivery care from professionals: 17.95% vs 34.81%). In addition, women with secondary or higher education, from urban residences and from the richest households were significantly more likely to utilize professional ANC and delivery services than women with no education, rural background, and poorest household, respectively. Maternal ANC utilization and delivery assistance from professionals varied as well by maternal age at first marriage, participation in deciding one’s own healthcare, order of birth and different region of the country.

Table 3 presents multilevel analyses of the relationships between maternal pregnancy intention and utilization of professional ANC and delivery care services. Compared to the single level logistic regression model without random effects, in multilevel models statistically significant differences were observed (LR chi-squared (2) = 60.56, p<0.01, for model ≥1 ANC from professionals; LR chi-squared (2) = 65.96, p<0.01, for model ≥4 ANC from professionals; LR chi-squared (2) = 63.55, p<0.01, for model delivery care from professionals), indicating the necessity of random effect models to quantify the relationship between maternal pregnancy intention and utilization of professional care services. The results showed that compared with mothers who had a wanted pregnancy, mothers who had an unwanted pregnancy were less likely to use professional antenatal and delivery care Mothers who married after age 18 years or more, had secondary or higher education, and were from richest households had higher odds of utilizing professional ANC and delivery care. However, utilization of professional ANC and delivery care was negatively associated with second or higher order births and rural residence.

**Table 3:** Association between maternal pregnancy intention status and use of professional ANC and delivery care, BDHS 2011.

Characteristics	Odds Ratio (95% CI)		
	≥1 professional ANC	≥4 professional ANC	Professional delivery care
**Intendedness at conception**			
Intended	1.00	1.00	1.00
Mistimed	0.82 (0.65–1.03)	1.06 (0.78–1.42)	0.98 (0.77–1.24)
Unwanted	0.69 (0.51–0.93)	0.56 (0.37–0.84)	0.70 (0.50–0.97)
**Individual**			
**Age of mothers, years**			
13–24	1.00	1.00	1.00
25–34	1.35 (1.06–1.70)	1.38 (1.04–1.82)	1.57 (1.23–2.02)
35–49	1.39 (0.92–2.09)	1.10 (0.64–1.89)	2.33 (1.42–3.85)
**Age at first marriage, years**			
<18	1.00	1.00	1.00
≥18	1.56 (1.24–1.95)	1.43 (1.11–1.84)	1.66 (1.31–2.11)
**Maternal education**			
No education	1.00	1.00	1.00
Primary education	1.72 (1.29–2.29)	1.38 (0.87–2.18)	1.37 (0.96–1.94)
Secondary or higher	4.63 (3.13–6.85)	3.65 (2.19–6.07)	3.07 (2.06–4.57)
**Age difference of the spouses, years**			
<5	1.00	1.00	1.00
5–10	1.10 (0.87–1.38)	0.87 (0.67–1.15)	0.99 (0.78–1.26)
≥11	1.28 (0.98–1.68)	0.97 (0.72–1.30)	1.00 (0.76–1.32)
**Deciding own health care**			
Participated	1.00	1.00	1.00
Not participated	0.93 (0.78–1.10)	0.92 (0.73–1.15)	1.01 (0.84–1.21)
**Current work status**			
Currently not working	1.00	1.00	1.00
Currently working	0.88 (0.65–1.18)	0.90 (0.61–1.31)	0.90 (0.66–1.22)
**Order of birth**			
First	1.00	1.00	1.00
Second and above	0.63 (0.50–0.80)	0.58 (0.44–0.76)	0.44 (0.34–0.57)
**Household**			
**Place of residence**			
Urban	1.00	1.00	1.00
Rural	0.58 (0.45–0.75)	0.37 (0.27–0.51)	0.44 (0.34–0.59)
**Socioeconomic status**			
Poorest	1	1.00	1
Poorer	1.25 (0.95–1.66)	1.28 (0.85–1.91)	1.11 (0.79–1.56)
Average	2.01 (1.49–2.71)	2.25 (1.47–3.42)	2.00 (1.45–2.76)
Richer	3.10 (2.20–4.36)	3.55 (2.23–5.64)	3.28 (2.25–4.79)
Richest	9.85 (5.95–16.31)	10.86 (5.73–20.58)	7.58 (4.47–12.83)
**Region**			
Barisal	1.00	1.00	1.00
Chittagong	1.02 (0.71–1.46)	0.57 (0.36–0.92)	0.83 (0.56–1.23)
Dhaka	0.96 (0.67–1.39)	0.64 (0.40–1.01)	0.80 (0.54–1.20)
Khulna	1.55 (1.02–2.36)	1.38 (0.85–2.26)	2.24 (1.45–3.46)
Rajshahi	1.50 (1.00–2.37)	0.78 (0.47–1.28)	1.26 (0.82–1.93)
Rangpur	1.25 (0.86–1.83)	1.90 (1.11–3.25)	1.30 (0.85–1.98)
Sylhet	0.84 (0.56–1.82)	0.45 (0.27–0.78)	0.68 (0.44–1.05)
**Variance (Cov) of random effect**			
Level 2 (Household)	1.09 (0.66)	1.66 (0.90)	0.96 (0.69)
Level 3 (Community)	0.51 (0.14)	0.81 (0.23)	0.61 (0.16)

ANC, Antenatal care; CI, Confidence interval

## Discussion

This study extends previous work on antenatal care utilization by assessing the effects of pregnancy intentions among women in Bangladesh. Using a nationally representative sample, our study shows that women who had unwanted or mistimed pregnancies were at greater risks of not utilizing professional antenatal care and delivery services compared to their counterparts who had planned pregnancies. The effect of unwanted pregnancy, however, was stronger on professional antenatal service utilizations than on the professional delivery care utilization. Furthermore, the risks for lower or nonuse of antenatal services were higher for younger, less educated, and poor women. Our results, therefore, highlight the importance of targeting these high risk groups of women to promote antenatal care and professional delivery care.

Consistent with previous studies showing associations between unintended pregnancy and inadequate antenatal care [16–18], our results confirmed that planned pregnancies and contraceptives should be promoted to reduce maternal and neonatal deaths in Bangladesh. Unwanted pregnancy may affect maternal ANC utilization from professionals through several mechanisms. Women who conceive an unwanted pregnancy might not recognize the pregnancy immediately [21]. Even if a woman suspects or knows she is pregnant, if the pregnancy is unwanted she may not seek antenatal care or follow good health practices out of fear that her pregnancy will be recognized by others, or even out of hopes that she will spontaneously abort [23, 41]. The aforementioned association might also be explained by maternal attitude and behavior; her feelings about having an unwanted pregnancy might contribute to conscious or unconscious neglect of her own health care. Another explanation could be women with unwanted pregnancy might get less familial or husbands support (in terms of economic or emotional) for her good health care during pregnancy. Women might also be emotionally and financially less prepared for an unwanted pregnancy and childbearing, resulting in less care for themselves and the developing unwanted fetus during pregnancy, as well [19].

We did not find any association between mistimed pregnancy and utilization of antenatal and delivery care from professionals. This result was unexpected but not surprising, since the feelings or lives of Bangladeshi women may not be as disturbed by a mistimed pregnancy compared with an unwanted pregnancy; its effect is not significantly different from those of intended pregnancies [19]. This finding is also consistent with a recent study in Turkey that shows no significant difference in antennal care use in mistimed and unintended pregnancies [17].

We found that women’s sociodemographic and household characteristics were associated with their use of professional antenatal and delivery care services. Women who married after reaching their 18^th^ birthday were more likely to seek antenatal care. This finding is consistent with previous studies showing that early marriage results in greater likelihood of unintended pregnancies [42], which is partially explained by younger women’s greater exposure to spousal violence [43] and lower utilization of professional ANC and delivery care services. Women with secondary or higher education utilize more professional antenatal and delivery care services than uneducated women, which could be due to educated women’s greater awareness of the benefits of antenatal services. This finding is supported by earlier studies in Bangladesh that suggest that maternal education is the strongest predictor of maternal care use [33]. Moreover, educated women tend to be more autonomous [44], hold more household decision-making power [45], and have more confidence and capability to make decisions regarding their own health care [46]. Higher utilization of professional antenatal and delivery care among women in the wealthiest households suggest that affordability could be an issue in antenatal care utilization. Although maternity services in the public sector are officially free in Bangladesh, several studies reported hidden costs to families (such as hospital fees and corruption) that may motivate poorer families to seek care from unqualified providers [47]. However, it is also possible that women in the wealthiest households are educated, have greater autonomy, and greater access to health information. Since pregnancy intentions are viewed as encompassing affective, cognitive, cultural and contextual dimensions [15], qualitative studies are needed to explore the pathways and potential mechanisms that may mediate the relationship between maternal pregnancy intention and antenatal and delivery care.

We found regional variations in antenatal care utilization. Women from Chittagong and Sylhet region were less likely to have an adequate number of professional ANC contacts than women from Barisal region. This may be because it is more difficulty to access professional care in the remote hill-tract areas in these regions. However, women from Rangpur region were significantly more likely to have an adequate number of professional ANC contacts, which may be due to the large-scale maternal and neonatal health programs that are being implemented in this economically impoverished region of Bangladesh [48]. Nonetheless, deliveries in Khulna region are significantly more likely to be attended by the professionals than those in Barisal. The reason for regional differences is unclear and needs further investigation.

Experiences of IPV, an important proximate determinant for utilizing reproductive and maternal health services, was not included in this study due to unavailability of domestic violence module in the 2011 BDHS. In an earlier study in Bangladesh using the 2007 BDHS, Rahman et al. found an association between maternal experience of IPV and low use of ANC and delivery assisted by a skilled provider. They argued that woman’s emotional and physical health might be affected by experiencing IPV which could discourage her from accessing appropriate maternal healthcare [7]. Moreover, as a means of control, a husband may prevent his wife from accessing healthcare [49]. Studies have also shown that women who experienced physical or sexual violence tend to have higher rates of unintended pregnancy, either because they are forced to have unprotected sex or because they are prevented from using contraception continuously by their violent spouse [10, 11, 13]. Women in abusive relationships might live in an environment where they have less control over their reproductive lives [13, 50]. These women, out of fear of escalated and/or continued violence, tend to submit sexually to their intimate partners, which may lead to unintended pregnancy [10]. Experience of unintended pregnancy itself is an important psychological and emotional stressor for a woman. When a woman experiences both unintended pregnancy and IPV she may be more likely to terminate a pregnancy [14, 51], and less likely to utilize ANC and delivery assistance from the professionals. Therefore, this study finding is unable to assess the possible effect of IPV on the relationship between maternal pregnancy intention and use of professional ANC and delivery care services in Bangladesh.

Our study has some limitations. First, due to retrospective reporting of pregnancy intention, there may be some recall bias in the data. Biases could also result due to unexpected pregnancy outcomes and experiences. To minimize recall bias and underreporting of unwanted births, we used information about the last-born children within the three years preceding the survey. Second, this study used data that measured maternal pregnancy intention only for pregnancies that ended in live births and thus overlooked pregnancies that were terminated early because of abortion, miscarriage, or stillbirth. These pregnancy outcomes are strongly associated with the experience of IPV [9, 11–14]. This study did not take into account the potential role of IPV due to lack of data. Finally, because of the cross-sectional design of the study the analysis can only provide evidence of statistical association, and cause-effect relationships cannot be inferred. Notwithstanding these limitations, our study has several strengths, including the large population based sample with national coverage, and the use of multilevel analysis considering probability weights, and clustering effects.

The findings of this study have important policy implications and far-reaching consequences for maternal care in Bangladesh. We suggest several priorities. First, since unintended pregnancy is associated with poor antenatal care utilization, policy makers should pay particular attention to promote effective and efficient use of contraceptives that directly reduce unwanted pregnancy. Making emergency contraceptives available could be a useful way to reduce unwanted pregnancy. Second, interventions for safe motherhood are needed to pay special attention to underprivileged and vulnerable women who may not access professional antenatal and delivery care [52, 53]. We recommend strengthening the demand-side financing (DSF) scheme that targets the bottom or the poorest 20% of the women. The DSF, launched in 2004, a maternal health voucher program developed by the Bangladesh Ministry of Health and Family Welfare (MOHFW) with support from World Health Organization (WHO), has been shown to improve access to maternal care [54]. Third, awareness needs to be created that lack of antenatal care may have harmful consequences not only for the fetus but also for the mother. In this regard mass media may play an important role.

## Conclusions

To our knowledge, this study is the first to document that, in Bangladesh, women with unintended pregnancies are at greater risks of lacking adequate antenatal and delivery care. Disadvantaged women particularly are at greater risks than women who are well-off. Bangladesh is striving towards SDGs 3 of reducing global MMR to fewer than 70 per 100000 live births by 2030 [55]. This study makes an important contribution by documenting unmet need for antenatal care, and the potential for unintended pregnancies to act as a significant barrier to the achievement of SDGs. Additionally, our findings are useful to inform governments, and local and international partners in other low-income countries who have been collaborating in the global effort to reduce maternal and neonatal deaths, of the need to focus on unintended pregnancies.
